# The Paradox of Ingestion of Dietary Cholesterol in “Vegans”

**DOI:** 10.3390/nu9070745

**Published:** 2017-07-12

**Authors:** Luiza Antoniazzi, Julio César Acosta-Navarro

**Affiliations:** Heart Institute (InCor), University of São Paulo, Medical School, São Paulo 03178-200, Brazil; jnavarro_2@hotmail.com

Recently, Clarys et al. [1] published a study comparing dietary intake of individuals following different dietary patterns (Vegan, Vegetarian, Semi-Vegetarian, Pesco-Vegetarian and Omnivorous Diet). The results showed that the vegetarian group presented a considerable consumption of dietary cholesterol, especially the vegan group (149 mg/day on average), which by definition does not eat animal foods. 

Considering that dietary cholesterol is limited to animal foods [2], we contacted the authors of the first study mentioned, who answered us promptly with some possibilities for such a finding. One explanation for this is that vegan products are not available in food composition tables, and using a similar product for the calculation (but not vegan) is overestimating the amount of cholesterol in the diet. Another possibility is the fact that other plant sterols are being counted, but these do not have the potential to raise plasma cholesterol and therefore should not be counted together with dietary cholesterol.

Looking for other studies in the literature that characterized the dietary intake of vegan individuals, we observed values lower than that reported by Clarys et al. [1] but higher than zero mg. Elorinne et al. [3] reported 44 mg and Schüpbach et al. [4] 12 mg for the vegan group. 

Carrying out a search for the term “vegan” in United States Department of Agriculture Food Composition Databases [5], we found 197 food items. The small amount of vegan foods analyzed and included in the food composition tables may be one more cause of difficulty in calculations, resulting in overestimation of the dietary cholesterol value. Considering this, the solution would be to calculate each recipe to dismember each ingredient, but it must be considered that it is not possible to dismember formulations from industrialized products, since this information is not supplied by the manufacturers, and researchers are limited to considering the information available in the nutritional information on food labels. 

Other methodological problem that could be responsible for this nutrient content differences are the criteria used in many studies to describe forms of vegetarian diets. There are studies in which lacto-ovo-vegetarian individuals consumed poultry and fish less than once every fifteen days [6], no more than once a week [7], or any type of meat less than six times per year [8].

A similar problem can arise with studies exclusively on a vegan group, because some people may declare themselves vegans but consume products that have been elaborated with some type of animal ingredient; it is necessary to apply some type of validation to determine whether these individuals belong to the vegan group.

Considering the importance of recognizing diets that are restricted in cholesterol in the context of a clear association to cardiovascular diseases, such as the vegetarian diet, and especially the vegan diet, which may, due to methodological issues such as nutrient calculation orders, not be correctly characterized, we believe that it is extremely important to discuss this issue in the scientific literature.
